# The continuum of care as a unifying framework for intergenerational and interspecies health equity

**DOI:** 10.3389/fpubh.2023.1236569

**Published:** 2023-10-04

**Authors:** Craig Stephen, Chris Walzer

**Affiliations:** ^1^McEachran Institute, Nanoose Bay, BC, Canada; ^2^Wildlife Conservation Society, New York, NY, United States; ^3^Research Institute of Wildlife Ecology, University of Veterinary Medicine, Vienna, Austria

**Keywords:** socio-ecologic, health, equity, interspecies, intergenerational, planetary, One Health

## Abstract

**Introduction:**

Unlocking the full potential of different people and organizations to address existential health threats requires shared goals and frameworks that allow people to see themselves contributing to a common and shared continuum of care. A new narrative to help people implement collective action for collective problems is needed.

**Methods:**

This paper is draw from the co-authors experience working from the local to international level on planetary health problems.

**Results:**

The proposed conceptual framework expands the socioecological model of health to help formulate multilevel approaches that foster healthier circumstances for all by revealing the mutual benefits that emerge from pooling expertise, funding, and political will to solve multiple problems with coordinated investment of resources and effort. It is intended to support program planning and communication. This framework is a response to the absence of systematic attempts to concurrently counteract the social and environmental conditions leading to disease, dysfunction and deficits which is increasingly seen as being problematic, especially as the root causes of health problems and solutions converge across species, sectors, and generations. The framework is embedded in the idea of interspecies and intergenerational health equity.

**Discussion:**

Ensuring interspecies and intergenerational health equity requires each actor to fulfill their roles along the continuum while supporting the needs of others. A socio-ecological continuum of care provides bundled options that combine knowledge from different sectors, disciplines and perspectives to guide interventions over time across a comprehensive array of services and support spanning all levels of needs, species and generations.

## Introduction

The entangled and reinforcing polycrisis of climate change, pandemics, food insecurity, biodiversity loss, pollution pressures and growing inequities amplifies the need to reform our perception of health systems. Recent reports have made it clear that there is a rapidly narrowing window of opportunity to preserve the social and environmental factors that secure health and resilience for all, and that time is not on our side, e.g., (1, 2). Integrated decision making must advance interspecies and intergenerational health equity – wherein steps taken to protect the health of one species today do not compromise the ability of future generations or other species to meet their own needs-if we are to secure the necessary capacities to remain well in rapidly changing social and environmental circumstances.

Governments, international forums, multilaterals, and civil society are increasing acknowledging that health, environmental issues and socio-economic drivers must be managed holistically across sectors and at multiple scales (3). The Quadripartite composed of the World Health Organization (WHO), Food and Agriculture Organization of the United Nations (FAO), United Nations Environment Program (UNEP), and World Organization for Animal Health (WOAH) is advocating for health systems that enhance intersectoral health governance and accelerate multisectoral coordination mechanisms.

While we have compelling data concerning climate change, pandemics, biodiversity loss, pollution and global inequities there remains insufficient action. Many areas of study and policy struggle with the reality that current approaches inadequately translate evidence at the rates and scales needed to inspire and sustain actions against global health threats. These include global health, One Health, EcoHealth, planetary health, and others. Ambiguities, uncertainties, and conflicting priorities make it hard to find the “best” way to mobilize knowledge into action. The growing spectrum of approaches can make it hard for people to determine where and how they can best contribute. Competition between approaches can obfuscate the critical guiding principle that society needs to recognize the intrinsic value of all living species for the health of humans, other animals, and ecosystems alike (4).

The socio-ecological model (SEM) of public health considers the physical, social, and economic determinants of health at both individual and population levels. It emphasizes the need for multiple interventions across diverse settings, from families to workplaces to government policies and services. The SEM was formalized as a health theory in the 1980s and is regularly used to examine the breadth of elements that influence manifestations and management of health outcomes (5). The SEM recognizes that individuals are embedded in a larger system, which must be improved for them to benefit from better health outcomes. The model encourages collaboration between stakeholders, including community members, organizations, policy makers and researchers to holistically address problems. It promotes the use of data from multiple sectors to gain a more complete understanding of an issue. An expanded SEM with a broadened continuum of care could be a coherent framework to help us shift from selecting from competing “right” approaches to fostering the right outcomes.

## An expanded socio-ecological model as a cross-cutting theme

Understanding and managing health from an interspecies point of view calls for awareness of similarities between the needs of different living things and limitations to meeting those needs in their shared setting. The SEM is a conceptual foundation used to protect health, ensure equitable and sustainable development and protect Earth’s biodiversity (Table 1).

**Table 1:** Socio-ecological thinking underpins health, sustainability, conservation and climate change adaptation.

Health is the cumulative effect of social and ecological factors that create threats, susceptibilities, resources, and capacities determining how well an individual, population, or ecosystem can cope with its lived reality. Health, whether for a person, pig, parrot, or place is determined by environmental, biological, economic, political, organizational, and cultural circumstances. Determinants of health enable access to resources for daily living and functioning, capacity to cope with change and stressors, and ability to meet expectations.
The United Nations’ sustainable development goals aim to end poverty, protect the planet, and improve the lives and prospects of everyone, everywhere. They are a shared blueprint for prosperity for people and the planet now and into the future. The sustainable development goals balance social, economic, and environmental sustainability, recognizing that action in one area will affect outcomes in others.
Biodiversity conservation involves the protection and management of species, habitats, ecosystems, and genetic diversity to sustain benefits for present and future generations. It equally considers socio-economic values and natural capital. Successful conservation requires coupling an integrated multidisciplinary understanding of the ecology of a region with a thorough understanding of human–environment interactions.
Climate change adaptation refers to adjustments in ecological, social and economic systems in response to climatic stimuli and their effects. Climate solutions for health and well-being must account for interactions between climate and socio-ecological systems to reduce vulnerability and generate climate-resilient development.

**Figure 1:**
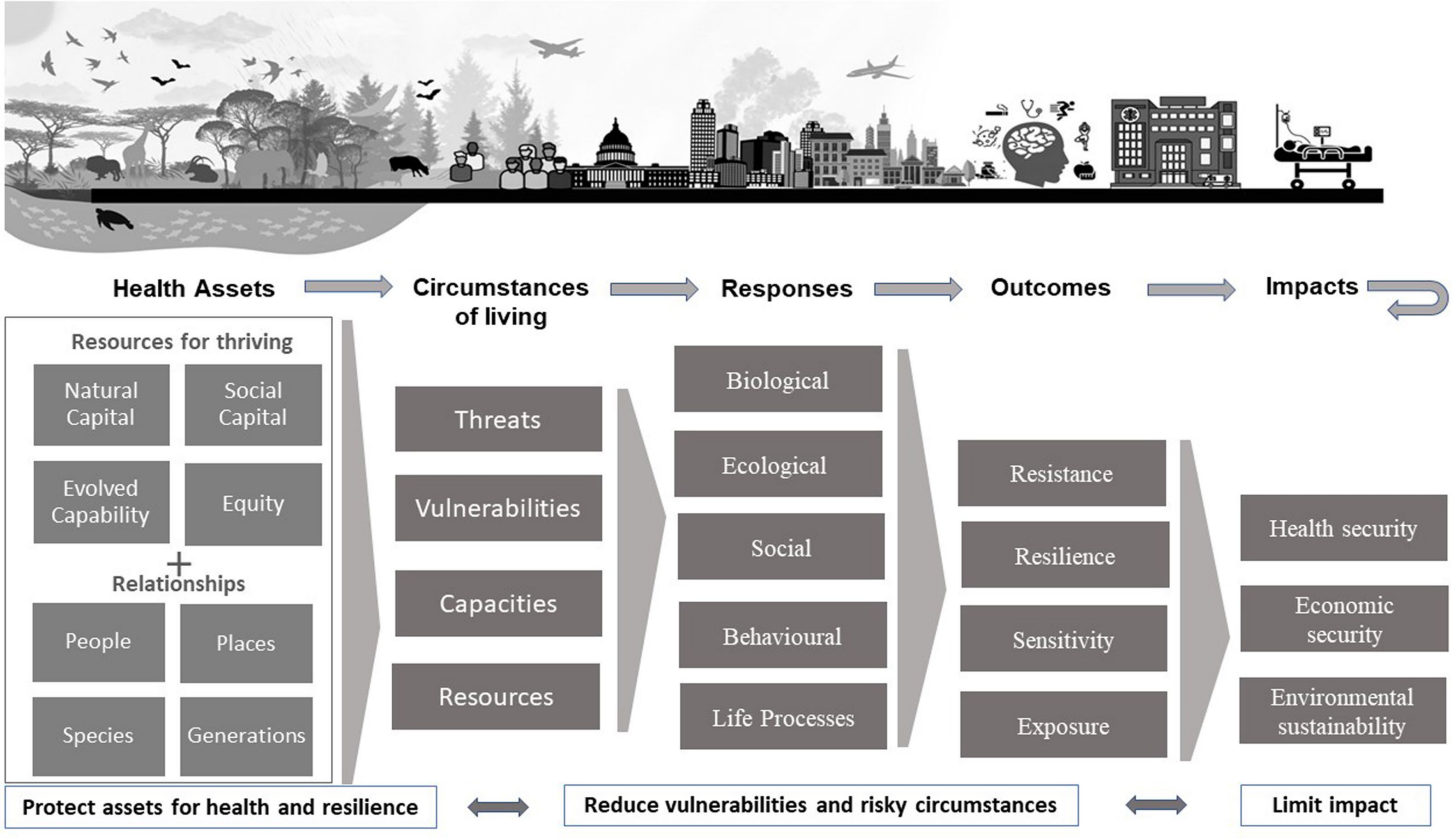
Generic socio-ecological model for health and resilience that is the basis for a socio-ecologic continuum of care. In this paper, we define social response as individual, collective, political and/or economic actions that make the quality of life and environment better or worse for those around them.

At its foundation, a SEM of health applicable to all living beings builds upon the networks of relationships among entities living in a particular setting, the setting’s natural assets, its environmental features and ecological relationships, and the behavioral mechanisms and physiological processes innate to the entities living in or using the shared setting (Figure 1). In this basic conceptualization, health equity across species and generations requires concurrent attention to the natural and social capitals that influence access to resources and capacities for health. Those capitals and resulting capacities are modulated by the circumstances of living and modified by interventions that affect the likelihood and impact of harms. SEM thinking can help us formulate multilevel approaches that foster healthier circumstances for all by revealing the mutual benefits that can emerge from pooling expertise, funding, and political will to solve multiple problems with a coordinated investment of resources and effort.

## Challenges to implementing an expanded socio-ecological policy in a coordinated manner

Despite accumulating evidence supporting the necessity to apply socio-ecological thinking to our major global challenges, there remain questions of how to operationalize this in policy and practice. There is a strong belief, built upon the Ottawa Charter on Health Promotion and amplified during the COVID-19 pandemic, that intersectoral approaches to health are essential to remedy significant problems (6). But opportunities to transfer this idea into the radical changes and practical solutions for global improvement of health have been missed (7). To date, there is sparse empirical evidence of effectiveness or impact of intersectoral approaches to health (8), in part due to the problem of attributing impact to specific efforts in a milieu where multiple parties are contributing (positively or negatively).

The partnerships needed to put socio-ecological thinking into practice (within or between organizations) are rarely unproblematic, especially where differences in power and resources exist and when interests are treated, funded, and managed separately (9). While the ideal of intersectoral health actions is great in principle, they can be hard to initiate, sustain and evaluate (10). Coalitions of interests supporting socially and ecologically integrated objectives have often been ephemeral, presenting a challenge to sustaining commitment to and involvement in long-term socio-ecological approaches (11, 12).

Challenges in identifying causal influences within complex socio-ecological systems and delays between interventions and outcomes can make it hard to convince policymakers and funders to invest in managing problems as a system. Policies that discount the future by emphasizing short-term economic growth and immediate returns on investments lead to inadequate investment in actions to address our polycrisis, since such expenditures may not pay dividends until years and generations later (2). Health policies routinely fail to adequately account for long-term impacts and costs, leading to a “discounting” of the future while concurrently failing to leverage the co-benefits of holistic approaches.

The preponderance of complex and wicked problems can hinder the development of policy solutions. Overlapping or competing roles and responsibilities raise concerns that policy will be ineffective in the face of many dynamic social and ecological drivers involving contradictory and incomplete interdependencies. A key obstacle to making progress with systems-level problems has been the tendency to act as if a “one-size-fits-all” approach will work (13), leading to debates as to which approach (e.g., Ecohealth vs. One Health) is best suited to which needs and problems. Rather than focus on differences in methods, it is more productive to focus on similarities in goals for resilience, equity, and the capacity to cope and adapt.

In every socio-ecological system, there is more than one species and more than one population. Each has its own unique requirements to be healthy and each can be subject to different expectations for their health. The socio-ecological goal is to combine knowledge, policies, and resources to make the setting healthier for all that live there, rather than addressing risks to only one group in a space shared with others. Interests and goals will, at times, conflict. When common goals cannot be identified or negotiated, programs need to function in ways that avoid creating or contributing to health inequities or limit potential to achieve complex interacting goals. They must strive for collaborations on solutions that lead to win-win-win scenarios for human, animal, and ecosystem health.

The COVID-19 pandemic is shaping substantial commitments and multilateral buy-in to promote holistic and integrated transformational change. A unifying framework that allows individuals and organizations to see how their skills, knowledge, and resources contribute to the cascade of circumstances that result in health will allow better assessment and valuation of the impacts of their own acts and their consequences across the health continuum. In doing so, people might be better able to appreciate the value of their contributions to interspecies and intergenerational health equity, to identify critical gaps, and to become empowered agents of transformational change.

## The continuum of care as an organizational framework for collective action

**Figure 2:**
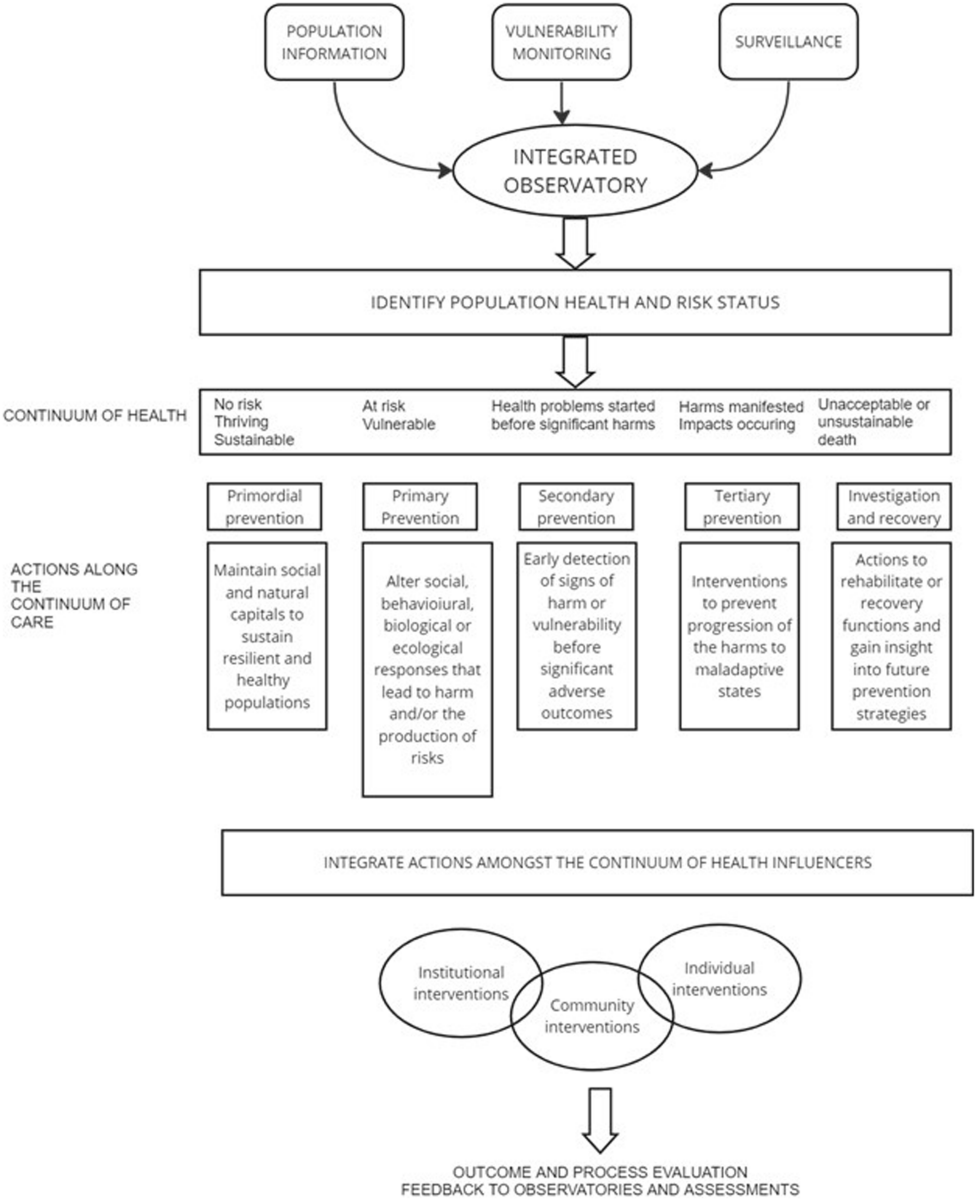
A generic socio-ecological continuum of care framework.

Health, (whether for individuals over time, between individuals in a population, or between populations in communities or species), exists along a continuum. A continuum of care is needed to manage the continuum of health (Figure 2).

To thrive, individuals, populations or species require a minimal set of resources, functions, and capabilities that enable them to cope with life’s challenges and meet expected endpoints. Society has traditionally looked to the health sectors to deal with deficits and disease. Resources have been heavily focused on health as a societal cost and on one-to-one interventions within the system, aimed largely at the restoration of health. A narrative that acknowledges health as a foundational societal asset is largely lacking. The absence of systematic attempts to concurrently counteract the social and environmental conditions leading to disease, dysfunction and deficits is increasingly seen as problematic, especially as the root causes of health problems and solutions converge across species, sectors, and generations.

Our proposed continuum of care (Figure 2) refers to bundled options that combine knowledge from different sectors, disciplines, and perspectives to guide interventions over time across a comprehensive array of services and support spanning all levels of need. Figure 2 builds from the widely used framework for putting population health theory and policy into practice (14).

Conceiving action on major global challenges along a continuum of care could make explicit one’s roles and contributions to progressing a common goal of health and sustainable socio-ecological systems without having to take responsibility for the entire system. A continuum of care could provide an overarching organizational framework for more integrated and interconnected actions on the social and ecological drivers of health and linked global threats.

Within any setting, there is not just one health, there are many. Different entities living in a shared setting can be at different places on their respective health continuum. An interspecies and intergenerational continuum of care considers the multiple, interacting components that influence the determinants of health and how they change over time and in different settings. Managing along the continuum requires the active, integrated involvement of diverse perspectives, disciplines, and sectors coordinated with the rights holders, stakeholders, and stewards of health. The goal is to make the setting healthier for all that live there now and in the future, rather than addressing risks to only one group currently in the shared space at the possible detriment of others. This can make sectors, decision-makers, and individuals more aware of the consequences of their actions.

The continuum of care could provide a seamless and coordinated course of multi-faceted actions designed to meet the health needs of individuals, populations or species as they move through life and face hazards, risks, and challenges. It ensures that actions meet populations “where they are” on the continuum of health, whether that be to maintain health, reduce vulnerability, reduce harms, or promote recovery.

Where any entity is on the continuum of health is a multi-faceted phenomenon with multiple ever-evolving causes and drivers. Knowing where they are is best achieved through partnerships that bring together different information and perspectives to characterize the nature of hazards and risks to which a population is sensitive and exposed, its capacity to cope with those threats, and its performance on specific health indicators. Integrated analysis of these observations helps strategically target interventions at the appropriate places along the continuum.

Interventions across shared land-and seascapes involving individuals (people, plants or animals), institutions (e.g., governments and civil society organizations) and communities are inevitably needed to identify and implement acceptable, feasible and sustainable interventions with lower likelihoods of unintended negative consequences and a higher likelihood of multi-solving impacts. Many of the disciplines needed to provide a broadened continuum of care fall outside of the usual realm of health practice and biomedical sciences. Although no single program can address the wide range of influences on health, the continuum of care approach helps orient programs away from more isolated and categorial approaches to more integrated ones. It illustrates the need for intersectoral activities and emphasizes the limitations of programs that do not consider the foundational upstream ecological processes nor the implications for action downstream in the continuum.

The continuum of care described above already exists to some extent but often in uncoordinated, unsustained, and unconnected ways that are not equitably distributed across countries, communities, or species. Without a cross-sectoral communication and engagement plan, it can be anticipated that the various actors along the continuum will work in isolation, inefficiently and, in some cases, at cross-purposes. The current global situation makes programs’ inefficiencies and conflicts intolerable. The current global situation also makes coordinated communication aspirational rather than operational. Inter-organizational and inter-sectoral partnerships are rarely without problems, especially where differences in power and resources exist, when there are overlapping or competing responsibilities, and when interests are treated and managed separately. A key step to progress is to recognize that a “one-size-fits-all” approach is ill suited to systems-based activities. The framework provided here serves as a conceptual foundation to support customized governance best suited to the settings and circumstances being managed.

The ideal of preserving resilient socio-ecological systems through a single integrated program should not be abandoned, but until systems science and systems governance becomes a norm (as opposed to the still largely siloed approach to research and policy), a more pragmatic view is needed; a view that recognizes that health exists on a continuum and that sustaining health requires actions across and along that continuum. There is a need to build capacity that supports collective actions of multiple organizations/individuals to ensure gaps in support and services needed across the health continuum can be addressed.

## Conclusion

Many researchers, health promoters, Indigenous leaders, and a growing number of policymakers understand the connections between the determinants of health across species and generations in shared settings. Progress on actions that concurrently work for health for all species and generations has, however, been frustratingly slow. Unlocking the full potential of different people and organizations to advance interspecies and intergenerational health equity requires shared goals that allow people to see themselves as part of and contributing to a common and shared continuum of care. Too often, in thinking about the role of collective action in health equity across species and time, individuals, governments and organizations are overwhelmed by sheer complexity and scale, reverting to a narrower focus on a specific problem. Such a view ignores how interconnected needs and solutions are across life stages and species within a shared setting.

A continuum of care perspective can help us recognize and strategically act upon the most pressing gaps in responding to and preventing disease and dysfunction while ensuring investment in the maintenance and restoration of health is not neglected, and that the full cost accounting of health benefits and associated costs to environment and climate are taken into account. While external agencies can catalyse, facilitate or support different individuals and organizations, ensuring interspecies and intergenerational health equity requires each actor to fulfill their roles along the continuum with consideration for supporting the needs of others.

The deep integration of knowledge, techniques, and expertise from multiple fields has been called the future of science and learning but evaluated examples of the application of this approach are lacking. The proliferation of socio-ecological systems-based approaches to health (such as One Health, EcoHealth, planetary health, conservation medicine, and health promotion) suggest that no single approach has been able to address all needs and problems and that no single case study can adequately illustrate the implementation of this framework. For example, an illustrative case using a settings-based approach from human health promotion may be insufficiently attentive to animal welfare or conservation goals, while still espousing its use of a socio-ecological perspective. To break silos of learning and doing, researchers, policy makers and practitioners need opportunities to roam across disciplines. They need to have chances to reflect on how their disciplinary framing of a problem affects their openness to innovative or disruptive opportunities by examining how their beliefs, judgments, and practices influence their approach to a problem. Unfortunately, attention to enhanced collaborations at high-level political fora has increased power struggles between dominant stakeholders, while investment in collaboration remains lacking (15). The objective of this paper has not to be to provide the solution to the problem. Rather it is our hope that the framework can serve to prompt inquiries and governance to improve coherence and collaboration in addressing health challenges and provide a common vision for research and actions that work towards interspecies and intergenerational health equity.

## Data availability statement

The original contributions presented in the study are included in the article/supplementary material, further inquiries can be directed to the corresponding author.

## Author contributions

CS contributed to the original conception of the framework and wrote the first draft of the paper. CW wrote sections of the manuscript. All authors contributed to the article and approved the submitted version.

## References

[1] IPBES. In: Brondizio ES Settele J Díaz S Ngo HT, editors. Global assessment report on biodiversity and ecosystem services of the intergovernmental science-policy platform on biodiversity and ecosystem services. Bonn, Germany: IPBES Secretariat (2019). 1148.

[2] Dasgupta P. (2021). The economics of biodiversity: the Dasgupta review. Available at: https://assets.publishing.service.gov.uk/government/uploads/system/uploads/attachment_data/file/962785/The_Economics_of_Biodiversity_The_Dasgupta_Review_Full_Report.pdf.

[3] Forman R Azzopardi-Muscat N Kirkby V Lessof S Nathan NL Pastorino G. Drawing light from the pandemic: rethinking strategies for health policy and beyond. Health Policy. (2022) 126:1–6. doi: 10.1016/j.healthpol.2021.12.001, PMID: 34961678 PMC8645287

[4] Mubareka S Amuasi J Banerjee A Carabin H Copper Jack J Jardine C. Strengthening a one health approach to emerging zoonoses. Facets. (2023) 8:1–64. doi: 10.1139/facets-2021-0190

[5] Kilanowski JF. Breadth of the socio-ecological model. J Agromedicine. (2017) 22:295–7. doi: 10.1080/1059924X.2017.1358971, PMID: 28742433

[6] Lefrançois T Malvy D Atlani-Duault L Benamouzig D Druais P-L Yazdanpanah Y. After 2 years of the COVID-19 pandemic, translating one health into action is urgent. Lancet. (2022) 401:789–94. doi: 10.1016/s0140-6736(22)01840-2, PMID: 36302392 PMC9595398

[7] Thompson SR Watson MC Tilford S. The Ottawa charter 30 years on: still an important standard for health promotion. Int J Health Promot Educ. (2018) 56:73–84. doi: 10.1080/14635240.2017.1415765

[8] Mikkonen JP. (2018). Intersectoral action for health: challenges, opportunities, and future directions in the WHO European region. PhD Dissertation. Available at: https://yorkspace.library.yorku.ca/xmlui/bitstream/handle/10315/35018/Mikkonen_Juha_2018_PhD.pdf?isAllowed=y&sequence=2.

[9] Amri M Chatur A O’Campo P. Intersectoral and multisectoral approaches to health policy: an umbrella review protocol. Health Res Policy Sys. (2022) 20:21. doi: 10.1186/s12961-022-00826-1, PMID: 35168597 PMC8845301

[10] Ollila E. Health in all policies: from rhetoric to action. Scand J Public Health. (2011) 39:11–8. doi: 10.1177/1403494810379895 20813799

[11] Margerum RD. Organizational commitment to integrated and collaborative management: matching strategies to constraints. Environ Manag. (2001) 28:421–31. doi: 10.1007/s002670010234 11494063

[12] Ribeiro CD van de Burgwal LH Regeer BJ. Overcoming challenges for designing and implementing the one health approach: a systematic review of the literature. One Health. (2019) 7:100085. doi: 10.1016/j.onehlt.2019.100085, PMID: 31016220 PMC6475629

[13] Alford J Head BW. Wicked and less wicked problems: a typology and a contingency framework. Polic Soc. (2017) 36:397–413. doi: 10.1080/14494035.2017.1361634

[14] Starfield B. Basic concepts in population health and health care. J Epidemiol Community Health. (2001) 55:452–4. doi: 10.1136/jech.55.7.452, PMID: 11413173 PMC1731926

[15] Spencer J McRobie E Dar O Rahman-Shepherd A Hasan N Hanefeld J. Is the current surge in political and financial attention to one health solidifying or splintering the movement? BMJ Glob Health. (2019) 4:e001102. doi: 10.1136/bmjgh-2018-001102 PMC6407571 30899558

